# The Grammatical Incorporation of Demonstratives in an Emerging Tactile Language

**DOI:** 10.3389/fpsyg.2020.579992

**Published:** 2021-01-13

**Authors:** Terra Edwards, Diane Brentari

**Affiliations:** ^1^Department of Sociology & Anthropology, Saint Louis University, St. Louis, MO, United States; ^2^Department of Linguistics, The University of Chicago, Chicago, IL, United States

**Keywords:** protactile, language emergence, deixis, demonstratives, intersubjectivity, tactile signed language, DeafBlind, tactile phonology

## Abstract

In this article, we analyze the grammatical incorporation of demonstratives in a tactile language, emerging in communities of DeafBlind signers in the US who communicate via reciprocal, tactile channels—a practice known as “protactile.” In the first part of the paper, we report on a synchronic analysis of recent data, identifying four types of “taps,” which have taken on different functions in protacitle language and communication. In the second part of the paper, we report on a diachronic analysis of data collected over the past 8 years. This analysis reveals the emergence of a new kind of “propriotactic” tap, which has been co-opted by the emerging phonological system of protactile language. We link the emergence of this unit to both demonstrative taps, and backchanneling taps, both of which emerged earlier. We show how these forms are all undergirded by an attention-modulation function, more or less backgrounded, and operating across different semiotic systems. In doing so, we contribute not only to what is known about demonstratives in tactile languages, but also to what is known about the role of demonstratives in the emergence of new languages.

## Introduction

In this article, we analyze the grammatical incorporation of demonstratives in a tactile language, currently emerging in communities of DeafBlind signers in the US who communicate via reciprocal, tactile channels—a practice known as “protactile.” We argue that this process is undergirded by reconfiguration of intersubjective relations, including habitual modes of attention to others and the environment. It is well known that deictic systems—and demonstratives in particular—are a powerful means of facilitating intersubjective coordination (e.g., Agha, 1994; Benveniste, 1971; Rommetveit, 1976; Duranti, 2010; Hanks, 2013; Sidnell, 2014; Evans et al., 2018a,b). However, in order to be effective, participants must assume reciprocal, perceptual access to each other and the environment. The systems we analyze in this article are emerging in DeafBlind communities where reciprocal modes of access have been re-organized around tactile channels (Edwards, 2015). In this article, we identify linguistic resources that have emerged since then for modulating attention within those newly re-contoured environments. In doing so, we contribute not only to what is known about demonstratives, but also to what is known about their role in the emergence of new languages.

## Background

Protactile language^1^ has emerged in groups of DeafBlind people who, for the most part, were born sighted, acquired American Sign Language (ASL) as children, and became blind slowly over several subsequent decades. As that process unfolded, visual communication in general, and ASL in particular, became increasingly untenable. Prior to the protactile movement, this was addressed via increased dependence on sighted interpreters. Since the inception of the protactile movement, there has been a politically and culturally framed demotion of visual communication and ASL, and an explicit push toward experimentation and innovation aimed at maximizing the potential of the tactile channel for purposes of communication (Edwards, 2014; McMillen, 2015; Granda and Nuccio, 2018; Clark, unpublished). As a result, new grammatical systems are beginning to emerge, which are optimized, as never before, to the tactile modality (Edwards and Brentari, 2020). As grammatical systems that interact most extensively with sensory-motor and interactional interfaces, phonology and deixis are at the center of this transformation.

In our prior research, we have shown that in roughly 10 years, a new phonological system has become conventional in protactile, DeafBlind communities, and that conventionalization of protactile phonology involves assigning specific grammatical roles to the four hands (and arms) of Signer 1 (“conveyer”) and Signer 2 (“receiver”) in “proprioceptive constructions” (PCs), which are comparable to “classifier constructions” in visual signed languages (Edwards and Brentari, 2020).^2^ In producing a PC, Signer 1 and Signer 2 work together to define the global space of articulation (similar to a “place of articulation”), within which, and to which, attention can be directed. We argue in what follows that the grammatical system governing the unfolding articulation of the PC incorporates and constrains protactile demonstratives. Protactile demonstratives are expressed using a combination of movement and contact that can be described as “tapping.” However, this is only one of several types of tapping that occur. In what follows, we trace the divergence of taps as they take on distinct functions in protactile language and communication.

### Demonstrative Categories

Diessel (1999) categorizes demonstratives according to their morphosyntactic properties from crosslinguistic and diachronic perspectives, and argues that demonstratives occur in four syntactic contexts (p. 1): (i) they are used as independent pronouns in argument position of verbs and adpositions (pronominal); (ii) they may co-occur with a noun in a noun phrase (adnominal); (iii) they may function as verb modifiers, and (adverbial); (iv) they may occur in copular and non-verbal clauses (identificational). Insofar as i–iv are distinguished formally, Deisssel assigns each to a corresponding grammatical category: (i) demonstrative pronoun; (i) demonstrative determiner; (ii) demonstrative adverb; and (iv) demonstrative identifier. He argues that the grammatical pathway demonstratives will take are determined by the syntactic context in which they occur (p. 2). In this study, we employ a modified set of Deissel’s demonstrative categories for several reasons. First, this study does not include elicitations designed to establish a noun-verb distinction (i.e., Abner et al., 2019). Therefore, we have replaced adnominal and adverbial demonstratives with a single category: “demonstrative modifier,” which can be applied either exophorically or endophorically, i.e., to refer to referents in the immediate environment, or to refer to linguistic aspects of the unfolding discourse. Second, we are tracking the diachronic development of a single form: “tap.” In the data we have analyzed here, tap does not appear in pronominal or identificational contexts. This reduces Deissel’s four categories to one: demonstrative modifier. The third reason we depart from Deissel’s categories is that our frame, by necessity, must handle an emerging (rather than well-established) linguistic system, and includes non-linguistic interactional signals, which, we argue, preceded, and contributed to the emergence of demonstratives with linguistic properties.

### Protactile Taps

In this article, we present evidence for four distinct types of taps: A tactile backchanneling tap, which is not part of the deictic system, but has an attention-modulating function, and may have functioned as a precursor to demonstrative and propriotactic taps; two kinds of demonstrative taps—one used for endophoric demonstrative reference and the other for exophoric demonstrative reference. In addition, we have identified a type of tap that is used to organize sequences of linguistic units by coordinating the four articulators of Signer 1 and Signer 2 (as discussed below). These forms, which we call “propriotactic” taps, are taps that have been co-opted by the phonological system, thereby entering the grammar of protactile language.

While only two of the four forms we analyze are demonstratives, we are interested in the intersubjective, attention-modulation function that underlies all four forms in more or less backgrounded ways. The order in which these forms emerge suggests a trajectory along which patterns in attention modulation, as part of a broader process of intersubjective coordination, are incorporated into, and integrated with, grammatical systems as those systems emerge. Tracking the way taps take on new functions in increasingly grammatical systems offers some insight into how this process can unfold, and helps us to understand the crucial role that demonstratives (and deixis, more generally) might play in that process.

The emergence and differentiation of protactile taps is part of a broader divergence between protactile language and ASL—the visual language on which it was originally scaffolded. Therefore, some background is needed on the relationship between the two languages.

### The Relationship of Protactile Language to ASL

Some DeafBlind people live as minorities within larger Deaf, sighted communities, while others are active members of a signing or non-signing DeafBlind community. Still others interact solely with hearing sighted people, and have no contact with Deaf or DeafBlind communities. Therefore, language and communication vary widely from community to community and across individuals. The dominant language in some DeafBlind communities in the United States is English, perceived via adaptive technologies such as amplification systems. In others, the dominant language is ASL. In order to perceive ASL through touch, the receiver places their hand(s) on top of the hand(s) of the signer to track the production of signs. Just as spoken languages require adaptive measures to be perceived by DeafBlind signers, adaptations and innovations are necessary for the perception of visual languages by DeafBlind signers as well. All of the participants in this study were fluent in ASL—whether perceived visually or tactually—prior to becoming DeafBlind, and hence can access ASL linguistic representations in some form.

**FIGURE 1:**
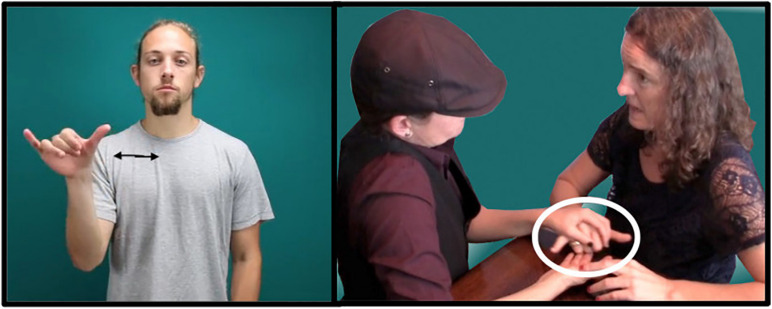
Handshape transferred to contact space via PC devices and conventions [ASL sign SAME (left) is re-produced from Hochgesang et al. (2018)].

However, for DeafBlind signers, ASL has the great disadvantage of being difficult to perceive, and therefore to *use*. According to DeafBlind, protactile leaders and educators aj Granda and Nuccio (2018), this difficulty is grounded in one, fundamental problem: the use of “air space,” or the space on and around the body of the signer. Protactile language is produced instead in “contact space,” or the space on the addressee’s body. This shift unlocks proprioception as a rich and accessible dimension of the tactile channel. In air space, locations are perceived relative to each other against a visual backdrop that is inaccessible for DeafBlind signers (e.g., “to the right of the mouth” vs. “to the right of the eye”). In contrast, locations in contact space can be clearly perceived against the proprioceptive backdrop of the listener’s own body. Figure 1 demonstrates this shift in the sign for SAME Figure 1; the citation form of the ASL sign is in Figure 1 (left). In Figure 1 (right) we see that both signers co-produce this sign. Signer 1 (right) produces an ASL “Y” handshape: 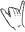 as in the ASL sign SAME; however, the sign is produced by making contact with Signer 2’s hand (left). The ASL handshape is not articulated in air space; instead, it has been transferred to contact space. All of the demonstratives analyzed in this paper occur in contact space.

In what sense, then, is this an emerging language of its own and not simply a variety of ASL? Edwards and Brentari (2020) have shown that the move to contact space triggered the emergence of new atomic units out of which signs are built, as well as new well-formedness constraints, which determine how protactile signs can and cannot be articulated. These constraints differ, fundamentally, from ASL. For example, instead of two hands, as in ASL, protactile language has four hands which can be activated in the creation of utterances. We label the four hands used in many protactile signs neutrally as “Articulators”: A1 (signer 1 dominant hand); A2 (signer 2 dominant hand); A3 (signer 1 non-dominant hand) and A 4 (signer 2 non-dominant hand). Each has its own set of specific linguistic functions, as Edwards and Brentari (2020) have described, and as we summarize in the section on proprioceptive constructions below.

In contact space, it is important that the addressee can feel signs as they are produced on their own body, that they can distinguish signs from one another, and that iconic and indexical grounds are maintained, linking signs, wherever relevant, to resonant and accessible tactile experiences, that can be shared by all speakers of the language. As reported by protactile signers themselves, the aim is not to preserve ASL to the greatest extent possible, but to embrace the potential of the proprioceptive/tactile modality for representing and evoking shared experiences. Granda and Nuccio (2018, p. 13) explain:

As Deaf children, we were drawn to visual imagery in ASL stories— transported into the vivid details of the worlds created for us. As DeafBlind adults, we still carry those values within us, but ASL doesn’t evoke those same feelings for us anymore. When you are perceiving a visual language through touch, the precision, beauty, and emotion are stripped away; the imagery is lost. […] If you try to access an ASL story through an interpreter […], you just feel a hand moving around in air space […]. In air space we are told what is happening for other people, but nothing happens for us.

This orientation suggests that protactile signers are prioritizing intuitive and effective communication over and against the preservation of ASL structures. Elements taken from ASL, such as “handshapes” are admitted into protactile language insofar as they meet these criteria. For example, there are two classifier handshapes for representing a “person” in ASL—the “1” handshape: 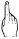 and an upside-down “V” handshape: 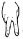. The “1” handshape 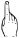 does not articulate to contact space easily because the bottom of the wrist is difficult to position and move on the body of the addressee in a precise and perceptible way. In contrast, in the “V” classifier 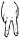, the two extended fingers representing the legs and the tips of the fingers make contact with the body. This handshape is perceptible. In addition, it can be modified for manner and direction of movement such that iconic and indexical grounds can be established and maintained in the unfolding of the communicative event. Given this, the upside-down V handshape is selected and the “1” handshape is discarded. In other words, only one of the two handshapes lends itself to the application of emerging, protactile constraints. This suggests that instead of working within the ASL grammar, and “adapting” or “compensating” as needed, protactile signers are operating within the new, tactile system, retrieving elements from ASL only insofar as they can conform to emerging patterns and rules. They are treating ASL as a kind of archival lexicon, or in the words of one participant, a “junk yard.” Furthermore, archived elements of ASL are only one source of material for building new protactile forms.

As we demonstrate in this article, another source of new protactile forms is interaction, and more specifically, cues that have emerged to facilitate intersubjective coordination, such as backchanneling and turn-taking (Edwards, 2014, p. 144--158)^3^. This paper is concerned with one type of form, which we refer to simply as “taps.” Anyone observing protactile communication for the first time will be struck by the sheer quantity of taps present in the stream of discourse, and will have some difficulty in interpreting those taps in ways their interlocutors seem to expect. In what follows, we argue that the complex multifunctionality taps have taken on can be traced back to a simple backchanneling cue used to signal continued attention or agreement. Backchanneling signals like these have been described in other DeafBlind communities (e.g., “YES-tapping” in Mesch, 2001; and see Willoughby et al., 2018, p. 9–11). We argue that these signals have been co-opted by protactile language to serve several different attention-modulating functions, including demonstrative reference.

### Pointing in Language Emergence

Typological and historical studies of language emergence are informative, but there are no cases of emergent spoken languages recent enough to be studied *al vivo*. Much of what we know about language emergence, then, comes from studying sign languages. In this growing body of work, the semiotic diversification and grammatical incorporation of pointing has become a focus (Meir, 2003; Coppola and Senghas, 2010; Pfau, 2011; Hopper and Traugott, 2003 [1993]; De Vos, 2014; Kocab et al., 2015; Mesh, 2017). As others have noted, grammaticalization has traditionally been studied in spoken languages and started with lexical forms, tracing how those forms take on new, grammatical functions as part of larger processes of language change (Hopper and Traugott, 2003 [1993]). However, there is growing interest in co-speech gesture and other forms of “visible action” (Kendon, 2004) as input to grammaticalization and related processes of language emergence in both spoken and visual signed language communication. This research has raised questions about how that input is treated by the linguistic system as containing structure that is accessible to the agents of language creation and language change. Deictic systems figure prominently in these debates.

For example, Kathryn Mesh, in a study examining the gestural origins of signs in San Juan Quiajije Chatino Sign Language, argues that “indicating” gestures are not, as McNeil (1992) has claimed, holistic “gesticulations,” but rather, forms with internal structure. For targets near the gesturer, elbow height is low and it increases as the distance of the target from the gesturer increases (Mesh, 2017, p. 65). This supports earlier findings (cited in Mesh, 2017, p. 47–48) that changes in the height of an indicating gesture correspond to the distance of the target among both hearing gesturers (Kendon, 1980; Levinson, 2003; Haviland, 2009; Ola Orie, 2009; Le Guen, 2011) and Deaf gesturers (van der Kooij, 2002; De Vos, 2014). Mesh shows that the internal structure of these indicating gestures is perceptible visually, without any access to the accompanying speech, and therefore constitutes rich input for creators of a new signed language (2017, p. 37–1122). Dachovksy (2018) argues facial expressions that are mutually accessible to signers of in the young Al-Sayyid Bedouin Sign Language serve as input to the creation of relative clause construction. In a similar vein, we are concerned with structured and meaningful resources available to the DeafBlind creators of protactile language. We focus here on two domains that are likely sources for such resources: ASL, which all of the people in this study acquired as children, and non-linguistic communication conventions that have emerged as part of broader patterns in protactile interaction.

While ASL is not perceptible enough to facilitate unimpeded communication among DeafBlind people (Reed et al., 1995), there are forms of knowledge that come with being a (former) speaker of ASL that are useful in creating a new language. For example, the intuition that “space” can be seized on for purposes of expressing grammatical relations. The concept of “air space,” as theorized by protactile signers, constitutes structured, input, which is then re-structured by the creators of protactile language, according to emerging principles, to yield “contact space.”

Another example: Former speakers of ASL are likely to have the intuition that new signs can be created via iconically motivated selection of some aspect of a referent to metonymically represent the whole (see Boyes-Braem, 1981, p. 42; Mandel, 1981, p. 204–211; Brennan, 1990, p. 11–36; Taub, 2001, p. 43–60). This pattern, too, can be transferred, at some level of abstraction, from visual to tactile domains.

ASL also provides a lexicon to the degree that lexical items are still cognitively accessible in individual speakers. These and other aspects of ASL are not raw materials, nor are they readily accessible as elements within a larger, structured system. Rather, they are *wrought products*—pieces of a language, now ill-suited to the world inhabited by its speakers. As protactile creators sift through the debris, they select elements that have affordances for communication in their new environment, this time, organized around tactile, rather than visual access. As that process unfolds, new patterns emerge that are different from the ones that had previously broken apart. These new patterns begin to govern what can and cannot be incorporated in the emerging system.

In addition to ASL (now sold for parts), protactile communicators draw on tactile communication conventions that emerged prior to, and operate beyond the bounds of, protactile language (Edwards, 2014). In this article, we focus on one of the many backchanneling cues that emerged and became conventional as part of that process: a repetitive tap, which is used to signal agreement or continued attention. We argue that these backchanneling cues have been drawn on in building a new deictic system that operates entirely via tactile channels.

Claims about grammaticalized pointing in signed languages tend to start with pointing gestures. In addition, space is often treated as the primary contextual variable driving semantic distinctions in the emergent system. Here, following the semiotic diversification of “taps” in protactile communication, we arrive at grammaticalized pointing not by way of pointing gestures or space, but by way of intersubjective attention modulators. While ego-centric spatial distinctions such as proximity to speaker, can, and often are, encoded in deictic systems, the heart of deixis is not space, but *access*. As Hanks (2009, p. 12) puts it: “The question is not, ‘Where is the referent?’ but ‘How do we identify the referent in relation to us?”’ The diachronic trajectory we trace in this article—from backchanneling cues to demonstrative modifiers, and from there, to more grammatical/functional units, reflects the idea that demonstrative reference is not grounded in spatial relations, but rather, in the multidimensional, intersubjective worlds within which pointing makes sense. It is not surprising, from this perspective, that interactional signals associated most closely with attention-modulation are precisely the kind of thing a protactile signer would intuitively seize on in building a new deictic system. This connection draws us away from thinking about language emergence as deriving from the grammaticalization of “space” (Coppola and Senghas, 2010) and instead shifts attention to the grammaticalization of intersubjectivity (Evans et al., 2018a,b, p. 113). From there, the richness of the semiotic input does not derive merely from the internal, structural features of a gesture or set of gestures available in the environment, but from any aspect of the environment that speaker and addressee can converge on as meaningful.

### The Grammaticalization of Intersubjectivity

In its most basic formulation, pointing is a mechanism for intersubjective coordination. The general tasks involved can be distributed over interlocking semiotic systems—such as grammatical systems, co-linguistic gesture, and interactional signals. Cross-linguistic research on spoken language has shown that deictic systems are particularly powerful in this regard, since they not only direct attention to objects of reference, but they do so according to diverse and conventional cues regarding what, where, and how to attend (Hanks, 1990; Diessel, 1999; Bühler, 2001 [1934]; Evans et al., 2018a). For example, Yucatec Maya offers speakers the option of signaling—by way of three distinct and conventional enclitics—that the referent is tactually, visually, or audibly accessible (Hanks, 2009, p. 14). Jahai, a language spoken in Malaysia, offers an “elevation” set, which includes distinctions such as “superjacent vs. subjacent,” i.e., located above the speech situation, as in “overhead, uphill, or upstream” vs. located below the speech situation, as in “underneath, downhill, or downstream” (Burenhult, 2003 cited in Evans et al. (2018a), p. 129, also see Forker, 2020). Each of these categories primes receptive attention in the addressee in its own, special way. Deictics, then, are a key resource, which can be drawn on by speakers and addressees to build up intersubjective access to, and knowledge about a shared world.

The protactile movement led to a radical re-configuration of human-human and human-environment relations (Edwards, 2018). As part of this, protactile signers learned to attend to one another and their environment in ways that were expectable to others, given no presumed access to visual channels (Edwards, 2015). In this article, we show how these new, reciprocal modes of attention are being enshrined in grammar. This case highlights the fact that deictics are not only tools for modulating attention; they also act as repositories for routine modes of access, including the channels along which attention can dependably be directed (Hanks, 2009, p. 22). Insofar as elevation or differences in visual, tactile, and auditory sources of information can function as organizing dimensions of life, they can become useful landmarks, and insofar as those landmarks are routinely referred to, they can be imprinted on the language as a system of choices for how to expediently orient the “searching perceptual activity” of one’s interlocutors (Bühler, 2001 [1934], p. 121). In other words, deictic systems anticipate the intersubjective worlds that shape them. The present study offers a glimpse of how that anticipatory capacity begins to develop in the earliest stages of language emergence, and the role demonstratives play. To this end, we begin with a brief summary of recent findings regarding the structure of proprioceptive constructions, which is necessary for understanding how taps are entering the grammar of protactile language.

## Proprioceptive Constructions

Expressions for events of motion and location have been an area of protactile grammar where a great deal of innovation has taken place, and in this section we describe some of the innovations that provide a backdrop for our analysis of demonstratives (further details can be found in Edwards and Brentari, 2020). In contrast to visual signed languages, where sign production involves the two articulators of the signer, protactile language has four potential articulators: the hands and arms of Signer 1 (the “speaker”) and the hands and arms of Signer 2 (the “listener”). The incorporation of the listener’s body as part of the articulatory apparatus yields a new kind of articulatory space, unattested in the world’s languages. Granda and Nuccio (2018) call this space “contact space,” and they distinguish this sharply from “air space,” used in visual sign languages such as ASL. In air space, locations are perceived relative to each other against a visual backdrop, such as the nose and eyes of the signer. In contact space, locations on the body of Signer 2 are activated and perceived against the backdrop of their own body.

**FIGURE 2:**
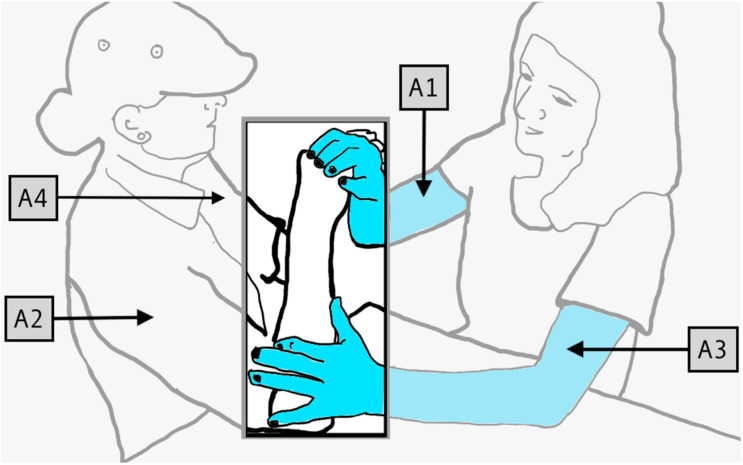
Four articulators used to produce PCs.

For example, in Figure 2, Signer 1 (right) is describing a lollipop to Signer 2 (left). The cylindrical stick of the lollipop is represented by the arm of Signer 2 (“A2”), as is the spherical candy portion. Their spatial relationship to one another is clear, since those relations are perceived by Signer 2, via proprioception, in the movements and positionings of their own body. Incorporating Signer 2’s body into the articulatory system unlocks great potential in the proprioceptive channel. However, it also generates a problem for the language: how can the articulators of Signer 1 and Signer 2 be coordinated in an efficient and effective manner?

One of the earliest stages in the emergence of protactile phonology resolves this problem by establishing conventional ways of signaling how and when Signer 1 wants Signer 2 to contribute to co-articulation of signs. Edwards and Brentari (2020) show that the conventionalization of such mechanisms involves assigning specific linguistic tasks to four articulators (“A1”–“A4” in Figure 2) in much the same way that the two hands in visual signed languages (“H1” and H2”) are assigned consistent and distinct tasks (Battison, 1978). A detailed account is available in Edwards and Brentari (2020). Here we provide a summary of those findings, which is required for understanding the synchronic and diachronic analysis of protactile demonstratives presented in this article.

Each PC includes at least one unit from each of the following categories, labeled according to their role in the larger construction: initiate (I), proprioceptive object (PO), prompt to continue (PTC), and movement-contact type (MC). These units, which are defined in Table 1, combine in the order given, to form a unified construction via rapid interchange between Signer 1 and Signer 2.

**TABLE 1:** Articulators and signing space in the protactile system.

**Articulatory components of Protactile Constructions**		
1	Articulator 1	Dominant hand—Signer 1
2	Articulator 2	Dominant hand—Signer 2
3	Articulator 3	Non-dominant hand—Signer 1
4	Articulator 4	Non-dominant hand—Signer 2
5	Contact Space	Locations on or near Signer 2’s body—“signing space” for protactile language.

### Initiate

**FIGURE 3:**
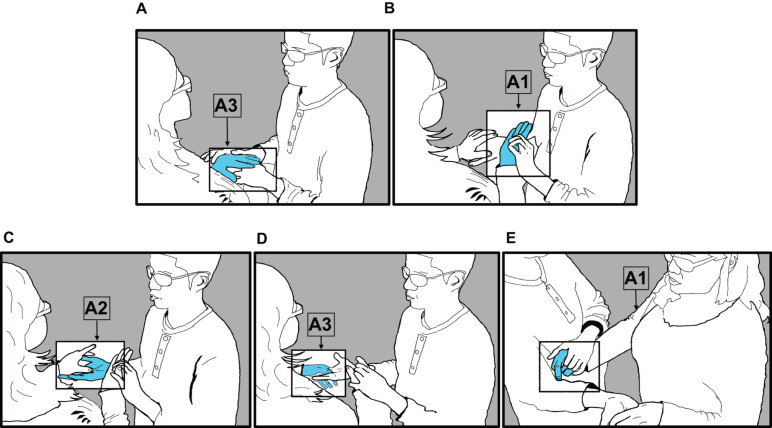
**(A–D)** Signer 1 (left), Signer 2 (right); **(E)** Signer 1 (right), Signer 2 (left). Proprioceptive Construction (PC) Units: **(A)** a general INITIATE form for the utterance; **(B)** a specific INITIATE form for the PC; **(C)** establishing the proprioceptive object (PO); **(D)** holding the PO in place for further specification (PTC), and **(E)** articulating a movement-contact unit (MCs).

In the temporal unfolding of the PC, the first to occur is “initiate”. As its name suggests, its function is to *initiate* a four-handed construction. There are seven attested types of initiate in the data we analyze here, one of which, is: “INITIATE-PROMPT-TAP”. In Figure 3A, Signer 1 (left) produces this form by tapping on the back of Signer 2’s (right) non-dominant hand. Signer 1 taps on Signer 2’s non-dominant hand (“A4”) with her non-dominant hand (“A3”). This alerts signer 2 that their active participation is required, and further instructions will follow. Attested initiates include: TOUCH, GRASP, MOVE, HOLD, TRACE, PO, PROMPT-TAP, AND PROMPT-PO.

### Proprioceptive Object

Once the PC has been initiated, a meaningful and phonologically constrained space, on which, or within which, further information can be conveyed must be established. We call that space, which is actively produced by Signer 2, the “proprioceptive object,” or “PO.” In Figure 3B, Signer 1 produces a second initiate, telling Signer 2 to select the PLANE PO. In Figure 3C, Signer 2 produces the PLANE PO using A2. The PO is significant for understanding demonstrative modifiers, because it generates the discourse-internal referents, to which endorphoric demonstrative modifiers refer. For example, once a PLANE PO, like the one in Figure 3C has been produced, Signer 1 can establish relations on the plane, and then tap on locations within it to foreground those locations. Attested POs include: PLANE (with sub-types: PENETRABLE, BENT, INCLINE); CYLINDER (with sub-type: TWISTED); SPHERE (with sub-type: PENETRABLE); INDIVIDUATED OBJECTS; and CONTAINER.

### Prompt to Continue

The third task in producing a PC is to maintain the active, contact signing space generated by the PO. It tells Signer 2, “Leave this hand here. There is more to come; or in the case of PUSH, relax this hand, we are done with it.” Therefore, we call this category of forms, Prompt-to-Continue (PTC). In Figure 3D, after Signer 2 has produced the requested PO (using A2), Signer 1 grips the PO (using A3) and holds onto it for the remainder of the PC. This gripping action is an example of a PTC unit. PTC is significant for understanding demonstrative modifiers, because it maintains the discourse-internal referents, to which endophoric demonstraive modifiers refer. It also maintains an active signing space, within which, demonstrative modifiers can be articulated. Attested PTCs include: GRIP, PENETRATE, PRESS, HOLD, and PUSH.

### Movement Contact Type

The fourth task in producing a PC is to draw attention to, and characterize, certain aspects of the PO, or a language-external referent, by producing tactile and proprioceptive cues that contain information about size, shape, location, or movement of an entity. These cues are called “Movement Contact Types,” or “MCs”. For example, in Figure 3E, Signer 1 (left) uses A1 (her right hand) to *trace* a line from the palm of A2 to the inside of the elbow. Figure 3E shows the end of a SLIDE describing a long, rectangular object. Attested MCs include: TRACE (with sub-type PO); GRIP (with sub-types TWIST, WIGGLE SLIDE, PULL, TRILL); SLIDE (with sub-type TRILL); PENETRATION; TAP (with sub-type TRILL); press (with sub-types WIGGLE and PO); SCRATCH; MOVE; and PUSH.

It is within this PC structure that taps can be differentiated formally and functionally. In the next section, after describing our methodology, we present evidence for establishing such distinctions, synchronically.

## Study Design and Procedures

In this article, we report on two studies. Study 1 is a synchronic analysis of the most recent data, collected in 2018. This analysis shows how taps function within, and are constrained by, protactile phonology. The results of this study will help orient the reader to the landscape of the current system. Study 2 zeros in on taps, tracing the different functions they take on over time, and how those functions change. This longitudinal study examines data collected at four moments in the emergence of the protactile language: In 2010, in the early stages of emergence; in 2015, 2016, and 2018. In the sections that follow, we include in-depth information about data collection, participants, procedures, stimuli, and transcription for each data set.

## Study 1 Methods

### Procedures

Recruitment took place in two phases. First, an email was sent to relevant community leaders explaining the project and requesting participation. That email was shared by them to a group of potential community members. A local DeafBlind educator then selected a subset of those who responded, based on her evaluation of high protactile proficiency. During data collection events, prior to filming, we gave consent forms to participants in their preferred format (e.g., Braille or large print). We also offered the services of qualified interpreters who could translate the consent form into protactile language. The first author, who is fluent in protactile language, offered to discuss the consent forms with each of the participants and answered questions/offered clarification, where requested. The consent forms included questions requesting permission to include images of these communication events in published research and other research and education contexts, such as conferences and classrooms. Once consent had been obtained, we commenced with data collection.

The 2018 data were generated in a description task, designed to elicit PCs. Data collection took place a privately owned home. Dyads of protactile signers were asked to stand next to a “cocktail” table—or a small, round table, which was tall enough to comfortably reach stimuli, while standing. Tactile landmarks were placed on the ground to signal locations where the cameras could pick up linguistic productions. The interactions were always between two protactile signers, both of whom were participants in the study. The stimuli were placed on the table in pseudo-random order and Signer 1 was instructed to “describe what they feel”. Signer 2 was told that Signer 1 would be describing something they felt. After the description, Signer 2 was offered an opportunity to feel the stimulus. After a certain number of stimuli, Signer 1 and Signer 2 changed roles. However, sometimes, after feeling the stimulus, Signer 2 chose to repeat a description with added changes or feedback. One of the co-authors and one member of the research staff were present throughout the task to operate the video cameras and place stimuli on the table, but were only in tactile contact with the participants while placing stimuli. The cameras were attached to the ceiling, using hooks and wire, and pointed down toward the participants, in order to capture contact and motion between them.

### Participants

The six participants in this study (3 males, 3 females, ages 32–53) were all DeafBlind individuals, who had participated in a protactile network for at least 3 years. The average number of years participating in a protactile network across the group was 6 years, and the range was 3–11 years. All but one of the participants were exposed to a visual sign language prior to age 5, via visual perception (those who became blind in adolescence or adulthood). One participant (who was born blind) had access to a visual sign language via tactile reception since birth. At the time these data were collected (2018), all six participants had been working full time in an environment where protactile language was in wide-spread use. Three of the participants also lived with protactile signers, and all of the participants attended informal protactile social events in the evenings and on weekends. In total, they reported an average of 49 h per person, per week, of protactile interaction and language-use. When asked what proportion of that time was spent with DeafBlind protactile signers, and what proportion was spent with sighted protactile signers (either hearing or Deaf), five participants said that most of their time was spent with DeafBlind protactile signers; One participant responded with irritation to the question, saying, “It doesn’t matter—the point is, they all know Protactile.” All but one of the participants (who reported growing up ambidextrous) reported that they grew up right-handed, but said that since being immersed in protactile environments, they now consider themselves more ambidextrous than they used to be.

### Stimuli

Proprioceptive constructions were elicited by presenting a series of tactile stimuli to the participants (Table 3). These objects were chosen because they were the same, or had corresponding characteristics as stimuli that were used in prior elicitation sessions, such as shape, size, or the presence of movable parts. Tactile stimuli like these offer opportunities for participants to convey information about motion and location events in protactile language, using real objects that can be explored tactually. The first two were presented as singular objects. The rest were presented in singular and plural conditions, as well as “multiple” conditions, which included a set of three, where two were the same, and one was different, along some dimension.

**TABLE 2:** Functional units of proprioceptive constructions and their associated articulators.

		**Functional Units of Proprioceptive Constructions**	**Articulator**
1	Initiate (I)	A request for active involvement of S2 in co-producing a PC	A1, A3
2	Proprioceptive Object (PO)	Active articulatory space- type selected in response to type of Initiate produced.	A2
3	Prompt to Continue (PTC)	Keeps selected articulatory space active for further information to be added.	A3
4	Movement Contact Type (MC)	Tactile and proprioceptive combinations of movement and contact that contain information about size, shape, location, or movement of an entity.	A1

**TABLE 3:** Stimuli for study one (2018).

**Stimuli**	**Conditions**
Large doll with braids and movable arms	Singular
Electronic braille display	Singular
Toy car ± self-propelled	Singular, plural, multiple
Lollipop	Singular, plural, multiple
Pen (± cap; ± ballpoint)	Singular, plural, multiple

In the case of the toy car stimulus, differences included size, shape, material, and whether or not the car was self-propelled (i.e., when you press it down, into a surface, and pull back, does it spring forward and travel out of reach? Or does it stay in place?). The lollipop stimulus involved differences in size and type of wrapper. In the case of the pen stimulus, the difference was whether the pen had a cap or was a ball point pen, where the ball point pop out when you press on the end of the pen with your thumb. In addition, some participants described the relative locations of each object on the table.

### Transcription

Descriptions of the stimuli were videotaped, labeled, and annotated using ELAN (Crasborn and Sloetjes, 2008). Coding one tier at a time, we identified the units produced by each articulator. In order to identify units of analysis within PCs, we assigned Signer 1 and Signer 2 independent tiers. Recall that Signer 1 is the principal conveyer of information. Signer 2 contributes to the articulation of the message, but in terms of information, is the principal receiver. In visual signed languages, the dominant hand (H1) and the non-dominant hand (H2) are assigned complementary roles; H1 is more active than H2 (Battison, 1978). In protactile language, four anatomical structures are available for producing each sign, which we assign to roles based on the degree to which they are active in linguistic productions as described in Table 1 : **A1** (dominant hand of Singer 1) > **A2** (dominant hand of Signer 2) > **A3** (non-dominant hand of Signer 1) > **A4** (non-dominant hand of Signer 2). Edwards and Brentari (2020) show that one of the earliest stages in the conventionalization of protactile phonology is the consistent alignment of particular articulators with particular linguistic functions, as described in Table 2: A1 is primarily responsible for producing MCs; A2 is primarily responsible for POs; A3 is primarily for producing PTCs; and A4 is rarely involved in linguistic production. As the present analysis highlights, A4 is least active in linguistic productions because its primary role is to track the movements of A1; Indeed, in these data contact between A4 and A1 is rarely broken. Its secondary role is to produce backchanneling cues. While A4 is tracking A1, simultaneous backchanneling cues can be produced by tapping on A1 with a few fingers, while maintaining a light grip with the remaining fingers. A2 can also produce backchanneling cues while performing other, linguistic tasks—though, as is discussed below, this is less common.

### Analyses

Analyses were aimed at identifying articulatory, distributional, and combinatory characteristics, which distinguish each of the taps from one another. To this end, we annotated each functional PC unit, including, but not limited to, taps on the tier corresponding to the articulator that produced it. In addition, we annotated taps produced outside of a PC unit, including taps on objects in the immediate environment, and taps functioning as backchanneling cues. We identified four types of taps, which we coded: I-PROMPT-TAP, MC-TAP, EX-TAP, and BC-TAP (Table 4).

**TABLE 4:** Tap functional units and coding labels.

**Coding Labels for TAP Functional Units**		
Propriotactic	Instruction by S1 to S2 to activate A2 for purposes of articulation, and/or that a “prompt-PO” is coming next	I-PROMPT-TAP
Demonstrative modifier	Draws attention to some aspect of a referent, or singles out one referent among others, in contact space	MC-TAP
Exophoric demonstrative modifier	Draws attention to some aspect of a referent, or singles out one referent among others, in the immediate environment	EX-TAP
Backchanneling signal	Signals S2’s continued attention and/or agreement	BC-TAP

In the sections that follow we outline our results and provide figures which contain the quantitative patterns found in the data; however, due to the small number of participants in these students, these results should be considered qualitative in nature.

## Study 1 Results and Discussion

### Phonological Characteristics

**FIGURE 4:**
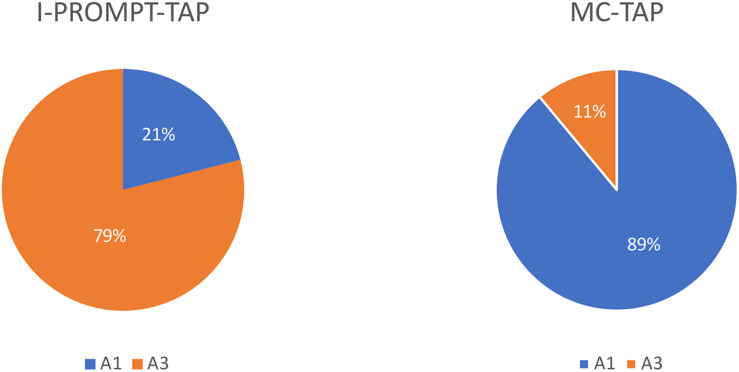
Articulatory-functional alignment in 2018 data.

One of the earliest stages in the conventionalization of protactile phonology is the consistent alignment of particular articulators with particular linguistic functions (Edwards and Brentari, 2020). This is, then, a potential resource for protactile signers for distinguishing the functional category to which they belong. For example, In the case of taps, I-PROMPT-TAP and MC-TAP belong to the INITIATE and MC categories, respectively. In the 2018 data, I-PROMPT-TAP and MC-TAP are associated with different articulators. I-PROMPT-TAP is produced with A3 most of the time (79% of 77 tokens), while MC-TAP is produced with A1 most of the time (89% of 392 tokens, Figure 4).

**FIGURE 5:**
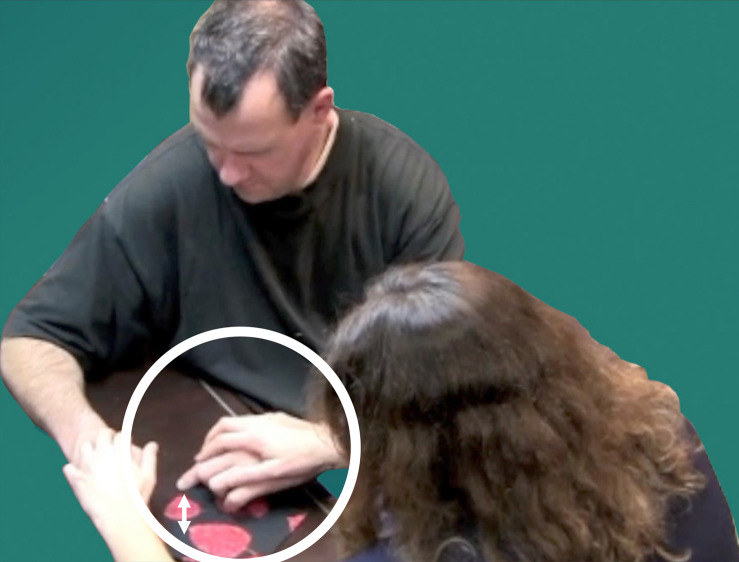
Signer 1 (left), Signer 2 (right). Signer 1 produces an exophoric demonstrative tap (A3) at the edge of a napkin.

Given this clear pattern in articulatory-functional alignment, the articulator used to produce the tap is one dimension along which demonstrative taps and propriotactic taps can be distinguished from one another. In addition, when a demonstrative modifier is used exophorically, its phonological characteristics change: it is not produced in contact space, i.e., on the body of Signer 2, with three articulators A1, A2, and A3. Instead, it is produced on an object in the immediate environment with a single articulator—either A1 or A3. For example, in Figure 5, Signer 1 (left) produces an exophoric demonstrative tap (A3) at the edge of the napkin, to indicate which edge he will fold next. The addressee (right) perceives the tap from a “listening position” (A4) but does not actively participate in articulation.

Finally, backchanneling taps can be distinguished from exophoric and endophoric demonstrative modifier taps and propriotactic taps, since they are produced by Signer 2 by either A2 or A4 (the two articulators, which belong to the “listener,” or Signer 2).

### Combinatory Patterns

**FIGURE 6:**
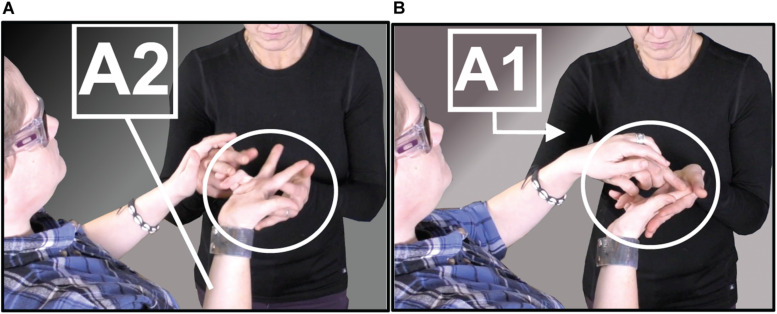
Signer 1 (right), Signer 2 (right). In **(A)**, Signer 2 produces PO-INDIVIDUATED-OBJECTS-3 (A2); In **(B)**, Signer 1 produces MC-TAP (A1) on the second of three individuated objects, to indicate position of referent in sequence.

Recall that propriotactic taps are a type of INITIATE, which function as an instruction from Signer 1 to Signer 2 to activate A2 for purposes of articulation and/or signal that another, more specific instruction will soon follow. We observe in these data that propriotactic (I-Prompt) taps cannot combine with other units to add information to that request. Signer 1 cannot, for example, produce the tapping motion with two fingers to signal that they are requesting two articulators instead of one. In contrast, demonstrative modifier taps can and do participate in various combinations. In addition, while propriotactic taps can only be used endophorically, demonstrative modifiers can be used alone (i.e., outside of a PC context) to refer, exophorically, to an object in the immediate environment (Figure 5, i.e., “this”), or they can combine with other meaningful elements in contact space to express information about number and/or location (e.g., “these two here” or “this here”); identify one item in a sequence of items (e.g., the second one of these three); information about shape (e.g. this cylindrical-thing”); or information about size (e.g., “this large one”). For example, in Figure 6A, Signer 2 (left) produces a PO representing three individuated objects (A2), after being prompted to do so by Signer 1. In Figure 6B, Signer 1 taps on the second of the three (A1), to indicate that the referent is second in the series of three.

### Distribution

Propriotactic (I-Prompt) taps occur at the beginning of a PC in the “initiate” slot, whereas the demonstrative modifier taps occur in the third position in a PC—the “MC” slot (Figure 3).

### Types of Taps in Protactile Language and Communication

Based on these differences in the 2018 data set, we propose the following types of taps in protactile language and communication:

(i) **backchanneling:** interactional signal for continued attention or agreement, is produced by Signer 2 using A2 or A4; occurs in various positions within, before, and after the completion of a PC; and cannot combine with other units.
(ii) **exophoric demonstrative modifier (Ex-tap)**: a TAP used to draw attention to, and add information about, a referent in the immediate environment; is produced most often with A1 and in all other cases, with A3; occurs outside of the PC context; and can combine with handshapes to indicate information about size, shape, and location.
(iii) **endophoric demonstrative modifier (MC-tap)**: a TAP used to draw attention to, and add information about, a referent in the unfolding discourse; is produced most often with A1 and in all other cases, with A3; occurs in the third position of the PC; and can combine with other PC units to convey information about size, shape, location, and movement of referent.
(iv) **propriotactic (I-Prompt)**: a TAP used to draw attention to, and request an action from, an articulator belonging to Signer 2; is produced most often with A3 and in all other cases, with A1; occurs in the first position of the PC; and cannot combine with other PC units.

While types i-iii are commonly found cross-linguistically in both signed and spoken languages, type iv---propriotactic taps---have, to our knowledge, never before been reported^4^. These forms are a type of Initiate, which function as a kind of “reception signal” (Bühler, 2001 [1934], p. 122). They tell Signer 2: “Be receptive here in this region of signing space—more information is coming.” For the phonological system, the presence of such a unit helps to optimize language to the tactile modality by shifting the ground of perception to the body of the addressee, where articulatory distinctions are made accessible through tactile channels, alone. In contrast, in visual sign languages, phonologically distinctive locations are perceived relative to each other against a visual backdrop that is inaccessible for DeafBlind signers (e.g., “to the right of the mouth” vs. “to the right of the eye”). Proprioceptive taps function as a conventionalized mechanism for activating the *listener’s* body, rather than the *signer’s* body, as the ground of perception Table 5. This new kind of unit, then appears to be specific to the tactile modality. In the remainder of this article, we examine the relationship of propriotactic taps to similar forms that emerged earlier in the roughly 10-year history of protactile language.

**TABLE 5:** Place of articulation for each type of tap.

**Type of tap**	**Sender**	**Place of articulation**
endophoric demonstratives	Signer 1	contact space often on a PO (Signer 2): A1
propriotactic taps	Signer 1	contact space often on a PO (Signer 2): A3
exophoric demonstratives	Signer 1	on an object in the environment
backchannel	Signer 2	contact space on Signer 1

## Study 2 Methods

Study 2 is a diachronic analysis of the emergence and conventionalization of the categories of taps identified in Study 1. Data include those from 2018, as well as those that were collected three times prior to 2018: in Seattle in 2010, early in the emergence of protactile communication practices (Edwards, 2014); in Seattle in 2015, as protactile practices were becoming conventionalized; and in 2016, when conventionalized practices were being transmitted from Seattle signers to a new group of DeafBlind students in Washington DC. Below, we provide information about the study design and procedures for each data collection event.

### Design and Procedures

The data used in this diachronic study were not collected in an identical manner, as is often the case when there are long intervals between data collection sessions, with changing linguistic and social circumstances. We describe each of the data sets we use in this study in the following sections.

### 2010 Data Collection

2010 recruitment procedures took place as part of a year-long period of sustained ethnographic fieldwork conducted by the first author. First, several meetings were held with relevant community leaders, in order to identify a context that would be appropriate for linguistic and interactional research. In those meetings, the community gave permission to the first author to videorecord a series of protactile workshops, where 11 DeafBlind signers and 2 instructors/organizers met twice weekly for 2.5 h, for a total of 10 weeks, in order to experiment with protactile communication in a range of activities. The workshops were held in a private room within a DeafBlind organization in Seattle.

Participants selected for the workshops by the DeafBlind instructors, were invited to discuss the research at length in individual meetings, prior to the start of the workshops, with Edwards. After those meetings, they made an informed decision of whether or not to participate. If they chose to participate, they were given a consent form in their preferred format (e.g., braille or large print). Edwards also offered to interpret the form into the preferred language of the participant. The consent forms included questions requesting permission to include images of interactions in the workshops in published research and other research and education contexts, such as conferences and classrooms. Once consent was obtained, Edwards and a team of videographers proceeded to videorecord approximately 120 h of interactional data generated during the 2010 protactile workshops, which were subsequently labeled, organized, and stored.

For the purposes of this study, we reviewed these data, looking for contexts that were maximally similar to the elicitation contexts created for the 2018 study. This included activities where objects, such as a tea strainer, a movable toy snake, or a phone charger, were being described by one DeafBlind participant to another; when objects were referred to as part of demonstrations/instructional activities, where one DeafBlind participant explains how to do something, such as use a crochet hook; and direction-giving activities—all of which were organized by the DeafBlind instructors. While these contexts were not elicitation contexts, and the objects introduced in the workshops were not framed as “stimuli,” we think these contexts offer an opportunity for comparison with the more targeted elicitations we conducted later.

### 2015 Data Collection

The 2015 recruitment procedures and consent process were identical to those described above for Study 1, and the consent forms included questions requesting permission to include images of interactions in the workshops in published research and other research and education contexts, such as conferences and classrooms. The data were generated by a description task, designed to elicit PCs using three stimuli: a lollipop, a jack (the kind children use to play the game “jacks”), and a complex wooden toy involving movable arms, magnets, and magnetized pieces. The first two stimuli were presented in a singular context (1 object) in a plural context (several of the same object in a row), and a “multiple” context (2 objects that are the same and one that differs in size, shape, or movability).

Data collection took place at a dining room table in a private home by both co-authors. Dyads of protactile signers were seated at the corner of the table. The interactions were always between two protactile signers, both of whom were participants in the study. They changed roles after a given item was completed, and discussed and gave feedback to one another about the clarity of a description, as it unfolded. We placed a cloth napkin with thick edges on the tabletop to provide a tactile boundary within which the stimuli would be placed. The stimuli were placed on the napkin in pseudo-random order and Signer 1 was instructed to “describe what they feel.” Signer 2 was told that Signer 1 would be describing something they felt. After the description, Signer 2, who was not exposed to the stimulus prior, picked up the object and explored it tactually. The co-authors were present throughout the task to operate the video camera, but were only in tactile contact with the participants when placing stimuli. The camera was on a tripod on the table, positioned above the participants pointing down, in order to capture contact and motion between them. In all cases, the dyads discussed aspects of the object and adjusted their descriptions—sometimes at great length. In addition, the stimuli had many different pieces and parts, each of which was described by the participants. Therefore, we collected a large number of tokens in response to a limited number of stimuli.

### 2016 Data Collection

The 2016 data we analyze were collected in two events. First, the two organizers of the 2010 Seattle workshops hosted a second set of protactile workshops in Washington, DC for a group of DeafBlind signers who were actively involved in local protactile networks. We analyze a series of interactions between 5 of the 7 participants, who took part in a direction-giving exercise. During this exercise, each participant was asked to give directions to locations within the building, to nearby buildings, and to other locations in the district. Workshops took place in an auxiliary classroom space at a local university. The second source of data from 2016 was generated in a description task, led by the first author, as well as a direction-giving task, similar to those that occurred in the workshops, also led by the first author. These data collection events took place in a lab on a university campus. The stimuli that were used in the description task included a soft block made out of fabric, a lollipop, and a jack. Two of the workshop participants were included in these sessions, along with one additional protactile signer. This yielded a total of 8 participants in the 2016 data set as a whole. 2016 recruitment procedures and consent were the same as those described above for the 2010 protactile workshops. However, instead of being invited to videorecord all of the sessions, the first author was invited to videorecord a subset of sessions, as determined by the group.

### Participants

There were a total of 15 participants in Study 2 (6 males and 9 females, ages 32–53 in 2018).

Thirteen participants were born sighted or partially sighted and acquired ASL as children via visual reception (all but one by the age of 7). 2 participants were born blind, and acquired ASL via tactile reception (both prior to the age of 7). 12 of the 15 participants were, at the time of data collection, immersed in protactile environments—at work, where protactile language was in wide-spread use, and/or in the evenings and on weekends, when they attended community events, or interacted with their protactile roommates. Three of the participants interacted with protactile signers somewhat often, according to their own reports, but with less frequency and consistency than the others, as they did not work in environments where protactile language was widespread. In Table 6, an X is placed under data collection event(s) for which each participant was present.

**TABLE 6:** Longitudinal participation.

	**2010**	**2015**	**2016a**	**2016b**	**2018**
Participant 1	X	X	X		
Participant 2	X	X	X		X
Participant 3	X	X			X
Participant 4	X				
Participant 5	X				
Participant 6	X				
Participant 7	X				
Participant 8		X			X
Participant 9		X			
Participant 10		X			
Participant 11		X	X	X	X
Participant 12			X	X	X
Participant 13				X	
Participant 14			X		
Participant 15					X

Participants 1 and 2 (Table 6) were leaders in the community, and took on the role of instructor or facilitator in the data collection events they were present for. They had been in close contact as colleagues since 2007 and during that period, developed a framework for thinking about tactile ways of doing everyday tasks, including communication. They hosted the 2010 workshops together in an effort to broaden their efforts across the community. From the outset, then, they had more experience with “protactile principles” (Granda and Nuccio, 2018) than any of the other participants. Participants 2–7 were exposed to protactile principles for the first time during the 2010 protactile workshops and were involved in the early innovations described below. Participants 8–15 who resided in either Seattle or Washington DC (or both at different times during the study) were exposed to protactile principles at least 1 year prior to their participation in their first data collection event, all via contact with protactile signers from Seattle.

### Transcription and Analyses

In a first pass, we located moments in each data set, when participants were asking questions such as “which one?” “where?” or moments where it seemed that the signer was trying to contrast one thing compared with another—e.g., if there were two chairs, and the signer was trying to draw attention to the one they wanted their interlocutor to sit in. We also looked at descriptions of objects with multiple parts, such as a lollipop (including the candy, the stick, and the wrapper), a series of such objects laid out on a table, and a series of such objects, where one differed in size, shape, or some other characteristic from the others. We identified moments in these descriptions when the signer foreground aspects of the object against a background (often paired with some characterization, i.e., “this [MC-TAP] + spherical thing [PO-SPHERE]”), or one object in a series against the others (i.e., “this one [MC-TAP] is the larger one”). We observed that MC-TAP was the most common form used in such contexts. We identified several additional taps, as well, which had related, but not identical functions: backchanneling taps (which we labeled “BC-TAP”), exophoric demonstrative modifier taps (which we labeled “EX-TAP”), and propriotactic demonstrative taps (which we labeled “I-PROMPT-TAP”).

We imported all video data (described above) into ELAN (Crasborn and Sloetjes, 2008). We created one tier for each of the four articulators (A1, A2, A3, and A4), and annotated each functional PC unit, including, but not limited to taps, on the tier corresponding to the articulator that produced it. In order to determine whether a form was in use, and how frequent its use was, we counted numbers of tokens per minute of active, transcribed, signing time. We also recorded the proportion of signing time spent in the “Signer 1” vs. “Signer 2” roles. Diachronic analyses, showing changes in the rate of occurrence of each category of taps is presented below.

## Study 2 Results and Discussion

**FIGURE 7:**
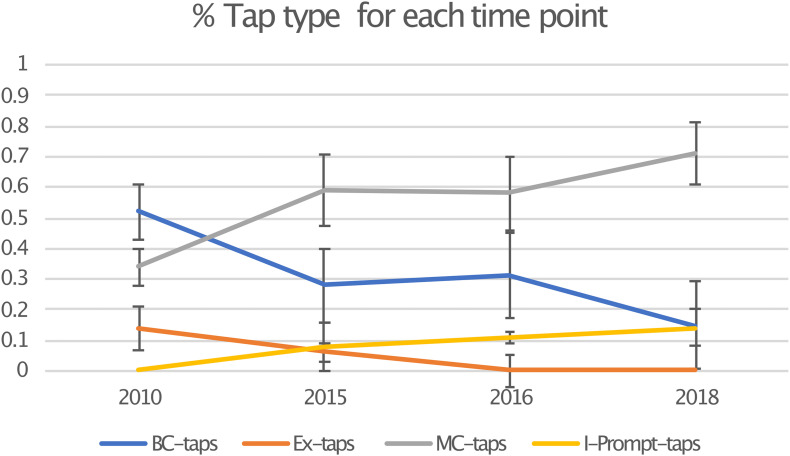
Semiotic re-distribution across categories (with standard error bars).

The results of Study 2 show that over time, the total number of taps produced by protactile signers are (a) becoming differentiated into a greater number of structural and semiotic types; and (b) being distributed differently across those categories in ways that optimize semiotic load (see Figure 7).

In the 2010 data, 483 taps were produced and in the 2018 data, 557 taps were produced. In 2010, backchanneling taps (“BC-taps”) were the most common, making up 52% of the total. Endophoric taps (“MC-taps”) were next, at 34%, followed by exophoric taps (“Ex-taps”), which accounted for 14%. Propriotactic taps (“I-Prompt-taps”) were not present in 2010 at all. From 2010 to 2018, we see the emergence and steady increase of propriotactic taps to reach 14% of the total by 2018. This coincides with a decrease in exophoric taps from 14% to 0. This suggests that a device for requesting the active participation of the addressee in sign production (i.e., the propriotactic tap), reduces dependence on exophoric reference.

In a parallel pattern, we see the proportion of endophoric (MC) taps increase from 34 to 71%, while backchanneling decreases from 53 to 15%. It may seem intuitive that an increase in affirmative backchanneling suggests an increase in understanding. However, early protactile communication was tenuous, and comprehension could not be taken for granted. Without conventionalized mechanisms for unlocking contact space, and without established modes of intersubjective access to the environment, consistent reassurance was necessary. As propriotactic taps and endophoric demonstratives emerged and became conventionalized, such frequent confirmation became far less necessary.

Of these four types of taps, we can see in Figure 7 that there are actually two pairs of taps that interact in terms of frequency, and, we would like to suggest, also in function. Backchannel taps and exophoric taps seem to lay the ground work for the two new types of taps, made possible by the proprioceptive construction that is argued for in Edwards and Brentari (2020). Both backchanneling taps and MC-taps index elements already present in the discourse, though of different types. Backchannel cues are responses to what was just said by the other person in the dyad, while MC-taps refer anaphorically or cataphorically to an element within the proprioceptive construction produced by the signer. This possibility derives from the conventionalized structure of the propriotactic construction: MC-tap (like all MCs) is interpreted in terms of its relevance to the preceding PO in the proprioceptive construction. In a parallel fashion, both exophoric taps and propriotactic taps introduce *new* information into the discourse, but, again, of different types. Exophoric taps introduce new entities from the surrounding environment. Propriotactic taps introduce new entities by way of new proprioceptive objects (POs).

**FIGURE 8:**
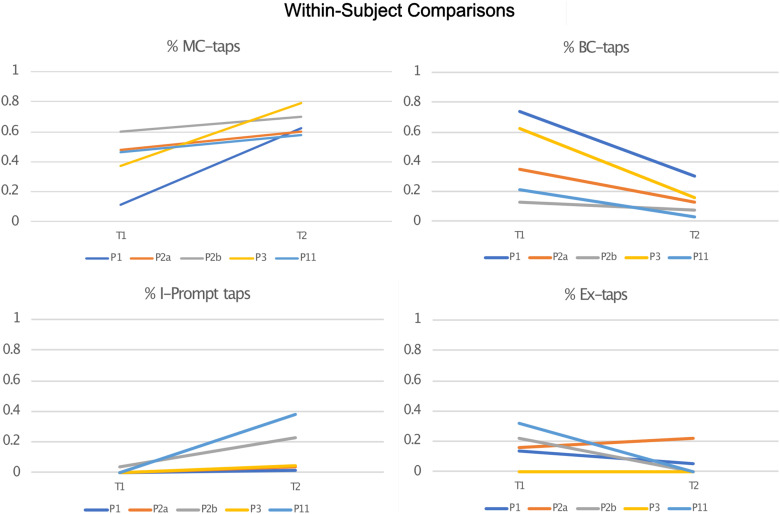
Within-subject comparisons of proportion of taps in each target group.

In order to analyze the emergence of MC-taps as compared to backchannel taps, and I-prompt taps as compared to exophoric taps, within-subject comparisons were carried out across two time points. For this analysis we compared individuals who were active in the dyad at least 20% of the time, and whose data were sampled at time points that were at least 2 years apart. Five of the 15 subjects^5^ met these criteria and are presented in Figure 8. In all within-subject comparisons, MC-taps and I-prompt taps increased over time, all Backchannel taps decreased over time, and in four out of five comparisons exophoric taps (one increased from 16 to 22%).

In analyzing these data, we were struck by the degree to which protactile signers struggled to communicate with each other in 2010. This often led to “checking” or “proving” that descriptions and instructions were accurate, by guiding one’s interlocutor to the aspects of the environment under discussion. By 2018, many of the mechanisms that were mere experiments in 2010 had become conventionalized, and therefore, there was a level of confidence in production, reception, and comprehension, which seemed to obviate strategies that involved excessive dependence on exophoric reference. We expect exophoric reference to remain available (despite its absence in the most recent data presented here). However, it is highly desirable for communicators to be able to give directions to a location in the immediate environment without walking the person to that location; It is also desirable to be able to describe or depict an object without having that object handy. It seems that these abilities were made possible by a process of semiotic redistribution across systems—I-Prompt taps are primarily organized by, and in service of, the linguistic system, broadly construed, while exophoric demonstratives are organized by, and in service of, an emerging deictic system. Over time, these systems have come to work in tandem, distributing attention-modulation tasks in ways the optimize the linguistic system to the intersubjective environment of protactile signers.

## Conclusion

In this study, we have followed the diversification and distribution of taps in protactile language. We have shown that backchanneling taps, which maintain continuity of attention across utterances, gain a new, related function in MC-taps, which use the developing linguistic system to maintain continuity of attention across related elements *within* a single utterance. Exophoric taps and propriotactic taps both introduce new entities into the discourse. Exophoric taps do so by directing attention to an object of reference in the immediate environment. This function then expands to include propriotactic taps, which introduce new entities into the discourse via proprioceptive constructions. The PC helps to optimize language to the tactile modality by incorporating the body of the addressee into the articulatory system, thereby making the proprioceptive sense available for purposes of perception. In doing so, it also offers a structure, within which, anaphoric and cataphoric reference can be reliably achieved.

Prior anthropological research has revealed the significant work protactile people have done to re-route reciprocal modes of attention through tactile channels, generating a new and re-contoured environment within which communication unfolds (Edwards, 2014). In this article, we have shown how protactile demonstratives are scaffolded on conventional backchanneling signals that emerged as part of, and were instrumental in, that process, and how from there, forms with more specialized grammatical functions began to emerge. In line with prior research, this supports the idea that deixis plays an important role in language emergence (e.g., Coppola and Senghas, 2010; De Vos, 2014; Kocab et al., 2015; Mesh, 2017). However, the path protactile language has taken, suggests a different basis for the connection. Coppola and Senghas (2010, p. 17) observe that “[g]rammaticalization processes need original forms on which to operate,” and following Heine et al. (1991) and Bybee (2003), they propose that “the sources for grammar are drawn from the most universal concrete and basic aspects of human experience, particularly the spatial environment and parts of the body.” The evidence presented here turns attention instead toward intersubjectivity as a potentially universal basis for grammaticalization. If languages are built under intersubjective pressures, we would expect that as a new language emerges, its grammar would develop sensitivities not only to space, but to whatever elements and relations are routinely and reciprocally accessible to its speakers.

## Data Availability Statement

The datasets presented in this article are not readily available in order to protect the confidentiality of the participants to the greatest degree possible. Requests to access the datasets should be directed to TE, terra.edwards@slu.edu.

## Ethics Statement

The studies involving human participants were reviewed and approved by IRB, Saint Louis University. The patients/participants provided their written informed consent to participate in this study. Written informed consent was obtained from the individual(s) for the publication of any potentially identifiable images or data included in this article.

## Author Contributions

TE is Assistant Professor of Anthropology at Saint Louis University, and has been conducting linguistic and anthropological research in DeafBlind communities in the United States for more than 14 years. DB is Professor of Linguistics at the University of Chicago and has written extensively on the phonology of signed languages.

## Conflict of Interest

The authors declare that the research was conducted in the absence of any commercial or financial relationships that could be construed as a potential conflict of interest.

## Footnotes

1 We use the term “protactile language” since this is the term that is currently in widespread use within the DeafBlind community that uses it.

2 Classifier constructions in sign languages are polymorphemic predicates, in which the movement is a verbal root (i.e., TO-GO or BE-LOCATED) and the handshape is an affix that represents a class of objects or size and shape of objects (Supalla, 1982; Zwisterlood, 2012).

3 We do not use the term “gesture” here to describe backchanneling. As Kendon (2004) notes, much of what would be considered gesture from one perspective might be considered language from another. There are too many conflicting uses of the term. We therefore identify backchanneling taps in terms of their primary communicative function—to modulate intersubjective attention in interaction.

4 As a general rule, it is assumed that turns at talk occur sequentially—that is, participants endeavor to speak “one-at-a-time” (Sacks et al., 1974, p. 696–735). Momentary deviations from this rule are permissible, for example, in the anticipatory completion of the other’s turn (Lerner, 1989, 1996), overlapping turns, as in assessments (Goodwin and Goodwin, 1987), and in collective turns, for example, when a group collectively greets a newcomer (Lerner, 1993; Schegloff, 2000). These activities display a greater range of interactional functions than backchanneling, and also are not seen by participants as problematic in the way that an “interruption” would be. Rather, they are treated as permissible, momentary deviations. In addition to these forms of overlap, speakers may grant “conditional access to the turn” (Schegloff, 2000, p. 5, and see also Lerner, 1996, p. 239). This occurs when a second speaker is invited to complete the turn of the first speaker, “conditional on the other’s use of that opportunity to further the initial speaker’s undertaking” (Schegloff, 2000, p. 5). For example, the “word search,” where the second speaker will be invited to produce the name of someone that the initial speaker is having trouble retrieving. This could be considered a self-initiated other-repair, since the speaker flags a source of trouble in the interaction and the addressee executes the correction (thanks to Simeon Floyd for pointing this out, personal communication). The phenomenon we are describing among protactile signers is most similar to conditional access to the turn, where the “invitation” to participate has become conventionalized in the initiate category of the proprioceptive construction. There are three conventional ways to “invite” the second speaker to co-produce signs— INITIATE-TOUCH, INITIATE-GRASP, and INITIATE-PROMPT. In addition, the production format (Goffman, 1981) is the same in cases of conditional access to the turn and PCs—while the animator role is distributed across two participants, the author role remains assigned to a single participant. However, there is one significant difference between conditional access and the co-construction of PCs: This collaborative construction of the utterance is not an exception to a general rule, it *is* the general rule—this is how protactile utterances are produced.

5 Participant 2 had two sessions that met the criteria for inclusion which are labeled Participant 2a and Participant 2b.
